# Prevalence of chronic kidney disease markers: Evidence from a three-million married population with fertility desire in rural China

**DOI:** 10.1038/s41598-017-02355-2

**Published:** 2017-06-02

**Authors:** Ye Du, Shikun Zhang, Mei Hu, Qiaomei Wang, Haiping Shen, Yiping Zhang, Donghai Yan, Yuanyuan Li, Man Zhang, Qun Meng

**Affiliations:** 1 Department of Social Medicine and Health Management, Public Health College, Harbin Medical University, Harbin, China; 2 Department of Maternal and Child Health, National Health and Family Planning Commission of the PRC, Beijing, China; 3 Department of Clinical Laboratory, Beijing Shijitan Hospital, Capital Medical University, Beijing, China; 4 Department of Maternal and Child Health Research, National Research Institute for Family Planning, Beijing, China; 5 Department of Statistics and Information, National Health and Family Planning Commission of the PRC, Beijing, China

## Abstract

We aimed to assess the prevalence of chronic kidney diseases (CKD) markers among the married residents with fertility desire in rural China. Demographic and clinical data were collected from the National Free Pre-Conception Health Examination Project. Estimated glomerular filtration rate (eGFR) < 60 mL/min//1.73 m^2^, proteinuria, and hematuria were defined as markers of CKD. GFR was evaluated by using serum creatinine level and the Asian-modified CKD epidemiology collaboration equation. Automated urine dry chemical and microscopic analyses were employed to identify proteinuria and hematuria. The prevalence of CKD markers was 2.92% in the 3,091,379 participants. eGFR < 60 mL/min//1.73 m^2^, hematuria and proteinuria was observed in 0.85%, 1.41% and 0.71%, respectively. The prevalence of CKD markers varied greatly across different geographical locations, which was the highest in the Eastern Region (3.86%; 95% confidence interval [CI]: 3.81–3.91%), moderate in the Central Region (2.80%; 95% CI: 2.77–2.82%), and lowest in the Western Region (2.62%; 95% CI: 2.59–2.65%). Hypertension, obesity, positive hepatitis B virus surface antigen (HBsAg), age (increased by every 5 years), female gender, and living area were potential risk factors for CKD. In rural China, the prevalence of CKD markers in the married couples with fertility desire is low.

## Introduction

The research on chronic kidney disease (CKD) has drawn more and more attention in the field of public health across the world, since the prevalence of CKD has been estimated to be high in many countries and regions^1–9^. The outcomes of advanced CKD can be devastating in terms of serious complications, demands for renal replacement therapy and increased risk for cardiovascular diseases^10–15^. The cost for CKD diagnosis and therapy rose up to $50.4 billion in the U.S. Medicare system in 2013^16^. The cost on CKD per capita per year was twice as high compared to the average Medicare patients^17^. CKD brings a heavy burden to patients and their families. Fortunately, CKD can be identified simply by blood and urine tests^18^. Timely diagnosis and treatment of CKD can help improve clinical outcome^19^.

According to Kidney Disease Improving Global Outcomes (KDIGO) Controversies Conference, the studies on CKD should include all ages and special populations^18^. In the past decade, research on CKD in China has been focused on urban residents^20–25^. In contrast, a very few study on CKD has been performed in rural areas, given China’s rural residents comprising 50.32% of its whole population^26^.

Poverty and social deprivation are emerging as major risk factors for CKD in both developing and developed countries^27^. There has been accumulating evidence suggesting that prevalence of CKD may dramatically increase in those populations with low socioeconomic status (SES)^5, 8, 9^. Living standard is relatively low in rural China due to dual economy. CKD in rural population of reproductive age has a heavy burden on their families and society.

The purpose of the current research is to evaluate the prevalence of CKD markers in the married couples with fertility desire across rural China, and to investigate variation in prevalence generated by age, gender, residential area, as well as common diseases including hypertension, obesity, diabetes and hepatitis B. The National Free Pre-Conception Health Examination Project (NFPHEP) has offered us a unique opportunity to explore the epidemiology of married population with fertility desire in rural China^28^. NFPHEP provided free pre-conception health examinations for married couples across 31 provinces and regions in rural China in 2010, in an attempt to improve the level of childbearing.

## Methods

### Design and participants

This research has been approved by the Institutional Review Board of Chinese Association of Maternal and Child Health Studies. All methods were performed in accordance with the relevant guidelines and regulations. A population-based cross-sectional study was conducted on the basis of NFPHEP database from January 1, 2010 to December 31, 2012. The random cluster sampling was used in this study by counties. The target people in the selected counties were eligible to participate into the study. 220 counties were sampled randomly across 31 provinces and regions in China. All couples with a plan to get pregnant within the next 6 months registered and completed a standardized family healthcare file in the village-level family planning service offices. They accepted a free medical examination by qualified medical care personnel according to the unified specification in the county-level family planning service organization. Each participant had written an informed consent before enrollment.

In order to assure the quality of examination, all the investigators and staff members had been well-trained on methodology and process. A manual of procedures was distributed to provide detailed instructions for how to administer questionnaires, test blood pressure, perform anthropometric measurements, as well as conduct biological specimen collection and transportation^28^. With the assistance of trained local health staff, all participants had completed a standardized questionnaire including social and demographic status (age, gender, career, education and residential region), personal health history (e.g., hypertension and diabetes). The data were submitted to and double checked by NFPHEP quality inspection center once every month.

The mainland of China was divided into three regions based on the level of economic development, which were Eastern Region (coastal areas), Central Region (inland areas) and Western Region (remote areas)^29^. According to their residential areas, all participants were divided into three regional groups: Eastern Region (including Liaoning Province, Beijing Municipality, Tianjin Municipality, Hebei Province, Shandong Province, Jiangsu Province, Shanghai Municipality, Zhejiang Province, Fujian Province, Guangdong Province, and Hainan Province), Central Region (including Shanxi Province, Jilin Province, Heilongjiang Province, Anhui Province, Jiangxi Province, Henan Province, Hubei Province, and Hunan Province), and Western Region (including Inner Mongolia Autonomous Region, Chongqing Municipality, Guangxi Zhuang Autonomous Region, Sichuan Province, Guizhou Province, Yunnan Province, Tibet Autonomous Region, Shaanxi Province, Gansu Province, Qinghai Province, Ningxia Hui Autonomous Region, and Xinjiang Uygur Autonomous Region)^29^. The age of the enrolled population was ranged from 20 to 49 years.

### Screening Protocol and Evaluation Criteria

According to the Kidney Disease Outcomes Quality Initiative (KDOQI) clinical practice guideline, estimated glomerular filtration rate (eGFR) < 60 mL/min//1.73 m^2^, proteinuria, and hematuria were defined as markers of CKD^30^. As our study did not repeat the measurements until three months later, the chronicity criterion of CKD was not applied to this definition. The morning spot urine samples were collected to identify hematuria and proteinuria by means of automated urine dry chemical analysis. Samples with positive hematuria for one or more times were reexamined through microscopic analysis within 2 hours by qualified technicians in the county clinical laboratories. Presence of 3 or more red blood cells per high-power field was considered abnormal. Subjects with hematuria and pyuria were considered to have urinary tract infection and excluded from data analysis. Women undergoing menstruation were also excluded from urine analysis.

Blood samples were collected by means of venipuncture after an overnight fast of at least 10 hours and immediately sent to the county clinical laboratories. Fasting blood glucose (only for women), serum creatinine (Scr), and hepatitis B virus surface antigen (HBsAg) were examined by qualified technicians. The reagent kits approved by the China Food and Drug Administration were chosen by the local laboratories on their preference, and were double checked by National Center of Clinical Laboratories for Quality Inspection (NCCLQI). Sensitivity, specificity, and κ value of the selected reagents from all the involved county laboratories were higher than 95%. Provincial Center of Clinical Laboratories for Quality Inspection carried out casual inspections and NCCLQTD conducted an external quality assessment (EQA) for quality control^31^.

Serum creatinine was measured by enzymic method with Enzymatic Creatinine-2 Reagents (Siemens Healthcare Diagnostics Inc.) and isotope dilution mass spectrometry (IDMS) method as the reference standard. Hepatitis B virus surface antigen (HBsAg) was detected by Siemens ADVIA 2400 Chemistry System with reagents produced by Abbott (Abbott Park, IL, USA) as the reference standard. eGFR was calculated by using Chinese modification of diet in renal disease (C-MDRD) equation^32^ and Asian-modified chronic kidney disease epidemiology collaboration (CKD-EPI) equation^33^, respectively as follows:

$$C-\mathrm{MDRD}:\,{\rm{eGFR}}(\mathrm{mL}/\,\min \,//1.73\,{{\rm{m}}}^{2})=175\times \,{\rm{Scr}}{(\mathrm{mg}/\mathrm{dl})}^{-1.234}\times {\rm{age}}\,{({\rm{years}})}^{-0.179}\times [{\rm{female}}\times 0.79];$$

$$\begin{array}{c}\mathrm{Asian} \mbox{-} \mathrm{modified}\,\mathrm{CKD} \mbox{-} \mathrm{EPI}\,\mathrm{equation}:\,{\rm{eGFR}}(\mathrm{mL}/\,\min \,//1.73\,{{\rm{m}}}^{2})=151\\ \quad \times \,{(0.993)}^{{\rm{age}}}\times \,{(\mathrm{Scr}/0.7)}^{-0.328}(\mathrm{female}:\,{\rm{if}}\,{\rm{Scr}}\le 0.7\,\mathrm{mg}/\mathrm{dl});\,151\\ \quad \times \,{(0.993)}^{{\rm{age}}}\times \,{(\mathrm{Scr}/0.7)}^{-1.210}({\rm{female}}:\,{\rm{if}}\,{\rm{Scr}} > 0.7\,\mathrm{mg}/\mathrm{dl});149\\ \quad \times \,{(0.993)}^{{\rm{age}}}\times \,{(\mathrm{Scr}/0.9)}^{-0.412}(\mathrm{male}:\,{\rm{if}}\,{\rm{Scr}}\le 0.9\,\mathrm{mg}/\mathrm{dl});149\\ \quad \times \,{(0.993)}^{{\rm{age}}}\times \,{(\mathrm{Scr}/0.9)}^{-1.210}(\mathrm{male}:\,{\rm{if}}\,{\rm{Scr}} > 0.9\,\mathrm{mg}/\mathrm{dl}).\end{array}$$

We adopted Asian-modified CKD-EPI equation to evaluate CKD prevalence.

Arterial blood pressure was measured in sitting position by sphygmomanometer three times at 5 minute intervals after the subjects had rested for at least 15 minutes. The mean of the three readings were calculated unless the difference between readings was greater than 10 mmHg, in which case the mean of the two closest readings was applied. Hypertension was defined as systolic blood pressure ≥140 mm Hg or diastolic blood pressure ≥90 mm Hg, or by any self-reported history of hypertension. Because fasting blood glucose and postprandial blood glucose of the men were not measured, for men, “diabetes” was defined as history of diabetes. For women, diabetes was defined as history of diabetes or fasting blood glucose ≥7.0 mmol/L. The body mass index (BMI) was calculated as weight in kilograms divided by height in meters squared. According to Chinese criteria of weight for adults^34^, 18.5 kg/m^2^ ≤ BMI < 24.0 kg/m^2^ was in normal range, BMI < 18.5 kg/m^2^ represented underweight, 24.0 kg/m^2^ ≤ BMI < 28.0 kg/m^2^ represented overweight. Obesity was defined as BM I ≥ 28.0 kg/m^2^.

### Statistical analysis

Continuous variables were analyzed by one-way ANOVA. Categorical variables were analyzed by χ^2^ test. The prevalence of CKD markers, eGFR < 60 mL/min//1.73 m^2^, hematuria and proteinuria were analyzed with age groups by spearman correlation test. To investigate the risk factors and indicators for CKD, logistic regression models were applied. The crude and multivariable adjusted odds ratios (ORs) were reported. Covariates included in the multivariable logistic regression models were hypertension (no *vs*. yes), obesity (no *vs*. yes), HBsAg positive (no *vs*. yes), gender (female *vs*. male), and region (eastern region: 0; central region: 1; and western region: 2). Age was defined as categorical variable with a 5-year interval, and 20–24 years was treated as a dummy variable in the Logistic Regression Model. All statistical data were analyzed after removing the missing items. The significant level was 0.05 and all of the analyses were two-sided tests. Statistical analyses were performed with SPSS version 21.0.

## Results

### Demographic and clinical characteristics of the studied population

3,091,379 participants filled in the questionnaire. Rate of loss of participants due to uncompleted blood test and urinalysis was 3.58%, 0.51% and 3.52%, respectively, in the Eastern Region, Central Region and Western Region. The average age was 27.04 ± 7.10 (standard deviation [SD]) years. As shown in Table 1, the proportion of female was 51.70%. The surveyed population mainly engaged in agriculture. Nearly 2/3 of people had junior middle-school education. The average level of Scr was 77.06 ± 20.35 µmol/L. 4.06% of the population was suffering from hypertension, with males more susceptible than females. More females had a history of diabetes than males (0.0214% *vs*. 0.0156%). The prevalence of diabetes was 1.41% for females. 5.64% of the population had positive HBsAg, with males more susceptible than females. The prevalence of underweight in females was 3 times higher than that in males, while the prevalence of overweight and obesity in males was twice as that in females.

**Table 1:** Sociodemographic and clinical characteristics of the married population with fertility desire in rural China.

Parameter	Total (n, %)	Male (n, %)	Female (n, %)	P-value
Participants	3091379 (100)	1493274 (48.30)	1598105 (51.70)	—
Age (years, mean ± SD)	27.04 ± 7.10	28.25 ± 8.68	25.91 ± 4.94	<0.001
Serum creatinine (µmol/l, mean ± SD)	77.06 ± 20.35	82.43 ± 19.76	72.05 ± 19.62	<0.001
BMI (kg/m^2^, mean ± SD)	21.91 ± 3.80	22.57 ± 3.70	21.29 ± 3.80	<0.001
EGFR by C-MDRD equation (mL/min//1.73 m^2^, mean ± SD)	109.46 ± 32.00	111.82 ± 30.87	107.25 ± 32.87	<0.001
EGFR by Asian modified CKD-EPI equation (mL/min//1.73 m^2^, mean ± SD)	110.84 ± 20.82	115.29 ± 18.97	106.68 ± 21.60	<0.001
Educational level				
Missing	26125 (0.85)	12282 (0.82)	13843 (0.87)	—
Illiterate	6455 (0.21)	1928 (0.13)	4527 (0.28)	<0.001
Primary school	147213 (4.76)	65180 (4.36)	82033 (5.13)	<0.001
Junior middle- school	2054914 (66.47)	975955 (65.36)	1078959 (67.51)	<0.001
Senior middle- school	546956 (17.69)	279105 (18.69)	267851 (16.76)	<0.001
University	302446 (9.78)	154755 (10.36)	147691 (9.24)	<0.001
Postgraduate	7270 (0.24)	4069 (0.27)	3201 (0.20)	<0.001
Profession				
Missing	49210 (1.59)	22875 (1.53)	26335 (1.65)	—
Farmer	2378085 (76.93)	1131701 (75.78)	1246384 (77.99)	<0.001
Worker	274242 (8.87)	152333 (10.20)	121909 (7.63)	<0.001
Attendant	109758 (3.55)	50795 (3.40)	58963 (3.69)	<0.001
Business people	72886 (2.36)	44507 (3.00)	28379 (1.78)	<0.001
House worker	28652 (0.93)	1774 (0.12)	26878 (1.68)	<0.001
Office clerk	114317 (3.70)	54320 (3.64)	59997 (3.75)	<0.001
Others	64229 (2.08)	34969 (2.34)	29260 (1.83)	<0.001
Hypertension	125600 (4.06)	79337 (5.31)	46263 (2.89)	<0.001
History of diabetes	575 (0.0186)	233 (0.0156)	342 (0.0214)	<0.001
Diabetes mellitus	22807 (0.74)	233 (0.02)	22574 (1.41)	<0.001
HBsAg positive	173860 (5.64)	94120 (6.32)	79740 (5.00)	<0.001
BMI (kg/m^2^)				
Missing	27889 (0.90)	13852 (0.90)	14037 (0.88)	
<18.5	269820 (8.81)	64637 (4.37)	205183 (12.84)	<0.001
18.5–24.0	2208312 (72.08)	1044120 (70.58)	1164192 (72.85)	<0.001
24.0–28.0	479556 (15.65)	304043 (20.55)	175513 (10.98)	<0.001
≥28	105802 (3.45)	66622 (4.50)	39180 (2.45)	<0.001
CKD markers (by Asian modified CKD-EPI equation)	90115 (2.92)	26996 (1.81)	63119 (3.95)	<0.001
GFR < 60 mL/min//1.73 m^2^ (by C-MDRD equation)	45110 (1.50)	14662 (0.98)	30448 (1.91)	<0.001
GFR < 60 mL/min//1.73 m^2^ (by Asian modified CKD-EPI equation)	26174 (0.85)	6410 (0.43)	19764 (1.24)	<0.001
Hematuria	43019 (1.41)	10923 (0.74)	32096 (2.03)	<0.001
Proteinuria	21578 (0.71)	9813 (0.66)	11765 (0.74)	<0.001
CKD markers (by Asian modified CKD-EPI equation and excluding hematuria)	47473 (1.54)	16126 (1.08)	31347 (1.96)	<0.001

CKD: chronic kidney disease. eGFR: estimated glomerular filtration rate. HBsAg: hepatitis B virus surface antigen. BMI: body-mass index. C-MDRD equation: Chinese modification of diet in renal disease equation. CKD-EPI equation: chronic kidney disease epidemiology collaboration equation. SD: standard deviation.

The prevalence of CKD markers by Asian-modified CKD-EPI equation for eGFR was 2.92% in the surveyed population (1.81% and 3.95%, respectively, in males and females). If hematuria was excluded, the prevalence of CKD markers was dropped to 1.54% (1.08% and 1.96%, respectively, in males and females). By adopting Asian-modified CKD-EPI equation for eGFR, the prevalence of eGFR below 60 mL/min//1.73 m^2^ was 0.85% (0.43% and 1.24% in males and females, respectively). By adopting C-MDRD formula, the prevalence of eGFR below 60 mL/min//1.73 m^2^ was 1.50% (0.98% and 1.91% in males and females, respectively). The prevalence of hematuria was 1.41% (2.03% *vs*. 0.74% in females vs. males). The prevalence of proteinuria was 0.71% (0.74% *vs*. 0.66% in females vs. males).

### The prevalence of CKD stages

As shown in Table 2, the prevalence of CKD 1–5 stage based on the estimated GFR by Asian-modified CKD-EPI equation were 1.66%, 0.40%, 0.79%, 0.03% and 0.03% respectively. There were differences between the prevalence of the males and the females in every CKD stage.

**Table 2:** Prevalence of CKD stages according to the eGFR in the married population with fertility desire in rural China by gender.

CKD stage	EGFR by Asian modified CKD-EPI equation (mL/min//1.73 m^2^)	Total (n, %)	Male (n, %)	Female (n, %)	P-value
1	≥90	51428 (1.66)	17766 (1.19)	33662 (2.11)	<0.001
2	60–89	12513 (0.40)	2820 (0.19)	9693 (0.61)	<0.001
3	30–59	24508 (0.79)	5669 (0.38)	18839 (1.18)	<0.001
3a	45–59	21235 (0.68)	4512 (0.30)	16723 (1.05)	<0.001
3b	30–44	3273 (0.11)	1157 (0.08)	2116 (0.13)	<0.001
4	15–29	794 (0.03)	416 (0.03)	378 (0.02)	<0.001
5	<15	872 (0.03)	325 (0.02)	547 (0.03)	<0.001

CKD: chronic kidney disease. eGFR: estimated glomerular filtration rate.. CKD-EPI equation: chronic kidney disease epidemiology collaboration equation.

### The prevalence of CKD markers in different geographical locations

As shown in Table 3, the prevalence of CKD markers varied greatly due to different geographical locations, which was the highest in the Eastern Region (3.86%), moderate in the Central Region (2.80%) and the lowest in the Western Region (2.62%). The prevalence of hematuria and proteinuria in Eastern Region was significantly higher than that in the Central and Western regions. Hypertension, obesity and positive HBsAg were most frequently observed in the Eastern Region.

**Table 3:** Prevalence of and risk factors for CKD in the married population with fertility desire by regions in rural China.

	Eastern region	Central region	Western region	P-value
Participants (n, %)	513318 (16.60)	1521785 (49.23)	1056276 (34.17)	
Women (n, %)	270297 (52.66)	785916 (51.64)	541892 (51.30)	<0.001
Age (years, mean ± SD)	27.74 ± 5.05	26.77 ± 8.61	27.10 ± 5.29	<0.001
BMI (kg/m^2^, mean ± SD)	22.10 ± 4.06	21.88 ± 3.41	21.86 ± 4.40	<0.001
CKD markers (by Asian modified CKD-EPI equation, %)	3.86 (3.81–3.91)	2.80 (2.77–2.82)	2.62 (2.59–2.65)	<0.001
EGFR < 60 mL/min//1.73 m^2^ (by Asian modified CKD-EPI equation, %)	0.85 (0.83–0.88)	0.89 (0.88–0.91)	0.78 (0.77–0.80)	<0.001
Hematuria (%)	2.31 (2.27–2.35)	1.29 (1.27–1.31)	1.14 (1.12–1.16)	<0.001
Proteinuria (%)	0.82 (0.79–0.84)	0.64 (0.63–0.65)	0.75 (0.73–0.77)	<0.001
Hypertension (%)	6.78 (6.71–6.85)	3.40 (3.37–3.43)	3.83 (3.79–3.86)	<0.001
Diabetes (%)	0.71 (0.69–0.74.)	0.87 (0.85–0.88)	0.56 (0.54–0.57)	<0.001
Obesity (%)	5.23 (5.17–5.29)	3.09 (3.06–3.11)	3.12 (3.09–3.15)	<0.001
HBsAg positive (%)	6.56 (6.49–6.63)	5.55 (5.51–5.58)	5.39 (5.35–5.43)	<0.001

Data are represented as n (%), % (95% CI), or mean ± SD.

BMI: body-mass index. CKD: chronic kidney disease. EGFR: estimated glomerular filtration rate. CKD-EPI equation: chronic kidney disease epidemiology collaboration equation. Obesity: BMI ≥ 28 (kg/m^2^). HBsAg: hepatitis B virus surface antigen. SD: standard deviation. CI: confidence interval.

### The prevalence of CKD markers in different age groups

**Figure 1:**
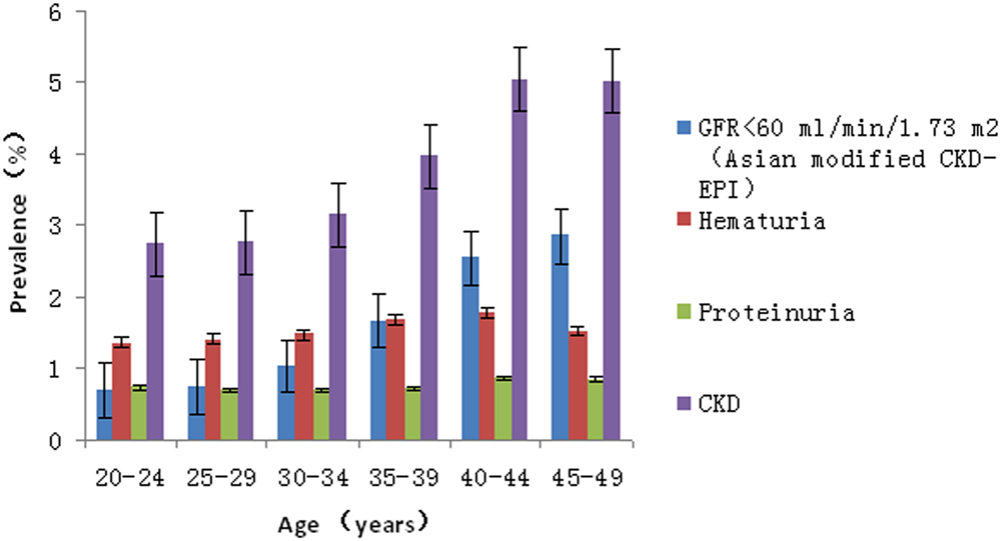
The prevalence of CKD indicators in the married population with fertility desire by age-groups in rural China.

As shown in Fig. 1, the prevalence of CKD markers except proteinuria increased with age. The prevalence of CKD markers in the aged 46–49 group was two times higher than that in the aged 20–25 group. The prevalence of eGFR below 60 mL/min//1.73 m^2^ (by Asian-modified CKD-EPI equation) in the aged 46–49 group was four times higher than that in the aged 20–25 group.

### The prevalence of CKD markers in the population with chronic diseases

As shown in Table 4, the prevalence of CKD markers was significantly higher in subjects with hypertension, diabetes, positive HBsAg, and obesity compared to their counterparts.

**Table 4:** The prevalence of CKD indicators of the married population with fertility desire by chronic diseases in rural China.

	CKD markers	eGFR < 60 mL/min//1.73 m^2^	Hematuria	Proteinuria
Hypertension (%)				
No	2.87 (2.85–2.89)	0.84 (0.82–0.85)	1.38 (1.36–1.39)	0.67 (0.66–0.68)
Yes	4.62 (4.51–4.74)	1.17 (1.11–1.23)	2.06 (1.98–2.14)	1.45 (1.39–1.52)
*p*	<0.001	<0.001	<0.001	<0.001
Diabetes (%)				
No	2.91 (2.89–2.93)	0.83 (0.82–0.84)	1.40 (1.39–1.41)	0.70 (0.69–0.71)
Yes	6.59 (6.26–6.91)	2.80 (2.58–3.02)	2.34 (2.14–2.53)	1.55 (1.39–1.71)
*p*	<0.001	<0.001	<0.001	<0.001
HBsAg positive (%)				
No	2.91 (2.89–2.92)	0.84 (0.83–0.85)	1.39 (1.38–1.41)	0.73 (0.72–0.75)
Yes	3.57 (3.48–3.66)	1.08 (1.03–1.12)	1.66 (1.59–1.72)	1.51 (1.35–1.67)
*p*	<0.001	<0.001	<0.001	<0.001
Obesity (%)				
No	2.90 (2.89–2.92)	0.84 (0.83–0.85)	1.40 (1.38–1.41)	0.66 (0.65–0.67)
Yes	4.14 (4.02–4.26)	1.02 (0.96–1.08)	1.76 (1.68–1.84)	5.63 (2.63–8.62)
*p*	<0.001	<0.001	<0.001	<0.001

Data are represented as % (95% CI).

CKD: chronic kidney disease. EGFR: estimated glomerular filtration rate. Obesity: body-mass index ≥28 (kg/m^2^). HBsAg: hepatitis B virus surface antigen. CI: confidence interval.

### The risk factors for CKD and indicators

Table 5 listed the crude and adjusted odds ratios (ORs) for CKD as well as potential indicators. Hypertension, obesity, positive HBsAg, female and age (increased by every 5 years) were independent risk factors for eGFR < 60 mL/min//1.73 m^2^. Hypertension, obesity, positive HBsAg, female, age (increased by every 5 years) and region were independent risk factors for hematuria. Independent risk factors for proteinuria included hypertension, obesity, positive HBsAg, female and region. Hypertension, obesity, positive HBsAg, female, age (increased by every 5 years) and region were independent risk factors for CKD.

**Table 5:** Risk factors for CKD and indicators of married population with fertility desire in rural China.

	OR (crude)				OR (adjusted)			
	CKD	eGFR < 60 mL/min//1.73 m^2^	Hematuria	Proteinuria	CKD	eGFR < 60 mL/min//1.73 m^2^	Hematuria	Proteinuria
Hypertension	1.36 (1.31–1.40)	1.41 (1.34–1.48)	1.51 (1.45–1.57)	2.17 (2.07–2.28)	1.63 (1.59–1.68)	1.36 (1.28–1.43)	1.55 (1.49–1.62)	2.04 (1.94–2.14)
Obesity	1.13 (1.08–1.17)	1.21 (1.14–1.29)	1.26 (1.21–1.33)	2.06 (1.96–2.18)	1.42 (1.37–1.46)	1.20 (1.13–1.27)	1.28 (1.22–1.34)	1.87 (1.77–1.98)
HBsAg positive	1.13 (1.10–1.17)	1.29 (1.23–1.35)	1.19 (1.15–1.24)	1.24 (1.18–1.31)	1.27 (1.24–1.31)	1.35 (1.29–1.42)	1.23 (1.19–1.28)	1.24 (1.18–1.31)
Female	9.53 (9.29–9.78)	2.91 (2.82–2.99)	2.79 (2.73–2.85)	1.13 (1.10–1.16)	2.44 (2.41–2.48)	3.40 (3.30–3.50)	3.00 (2.93–3.07)	1.16 (1.13–1.19)
Age (years)								
20–24	1.00	1.00	1.00	1.00	1.00	1.00	1.00	1.00
25–29	1.01 (0.99–1.03)	1.05 (1.01–1.08)	1.04 (1.02–1.07)	0.92 (0.90–0.95)	1.14 (1.12–1.16)	1.27 (1.23–1.31)	1.19 (1.17–1.22)	0.91 (0.88–0.94)
30–34	1.15 (1.13–1.17)	1.47 (1.42–1.52)	1.10 (1.06–1.13)	0.94 (0.90–0.98)	1.34 (1.31–1.37)	1.89 (1.82–1.96)	1.30 (1.26–1.34)	0.92 (0.88–0.96)
35–39	1.47 (1.43–1.51)	2.37 (2.28–2.47)	1.24 (1.19–1.29)	0.98 (0.92–1.04)	1.72 (1.67–1.76)	3.02 (2.90–3.15)	1.50 (1.44–1.56)	0.95 (0.89–1.01)
40–44	1.89 (1.81–1.96)	3.67 (3.47–3.88)	1.33 (1.25–1.42)	1.17 (1.07–1.29)	2.23(2.15–2.32)	4.78 (4.51–5.06)	1.64 (1.54–1.75)	1.12 (1.02–1.23)
45–49	1.87 (1.73–2.03)	4.06 (3.65–4.52)	1.13 (0.98–1.30)	1.14 (0.94–1.38)	2.59 (2.39–2.81)	6.66 (5.97–7.43)	1.67 (1.44–1.92)	1.09 (0.90–1.32)
Region								
Eastern Region	1.00	1.00	1.00	1.00	1.00	1.00	1.00	1.00
Central Region	0.72 (0.70–0.73)	1.05 (1.02–1.09)	0.55 (0.54–0.57)	0.78 (0.75–0.81)	0.76 (0.74–0.77)	1.14 (1.10–1.18)	0.59 (0.57–0.60)	0.82 (0.79–0.85)
Western Region	0.67 (0.66–0.68)	0.91 (0.87–0.94)	0.49 (0.48–0.50)	0.92 (0.88–0.95)	0.72 (0.71–0.73)	0.99 (0.96–1.03)	0.52 (0.51–0.54)	0.98 (0.95–1.02)

Data are represented as odds ratio (95% CI).

CKD: chronic kidney disease. EGFR: estimated glomerular filtration rate (by Asian modified CKD-EPI equation). Obesity: body-mass index ≥28 (kg/m^2^). HBsAg: hepatitis B virus surface antigen. CI: confidence interval.

## Discussion

This study of three-million participants provides important information on CKD in rural China and fills the gap in knowledge. The prevalence of CKD markers (2.92%) among married population with fertility desire in rural China is markedly lower than the average national level in Mainland China (10.8%) and in Taiwan (11.93%)^5, 22^. A higher prevalence of CKD markers in the developed Eastern Region is different from other studies in which the prevalence of CKD was higher in people with low socioeconomic status. The factors below may account for the difference. First, age variation exists in the studies. The average age in this study, in Mainland China and in Taiwan was 27.04 ± 7.10, 49.6 ± 15.2, and 41.8 ± 14.1 years, respectively^5, 22^. Age is an independent risk factor for CKD. Second, the prevalence of risk factors for CKD diverges greatly in different investigations. Participants in Mainland China study were characterized by an incidence of hypertension as 35.4% and self-reported diabetes as 7.4%^22^. The prevalence of hypertension and diabetes reported by the study in Taiwan was 20.7% and 5.4%, respectively^5^. Those values outnumber the corresponding ones in our study. A sampling bias stemming from more subjects with chronic diseases may result in higher prevalence of CKD in some previous studies. Third, different methods were applied for eGFR assessment in different studies. The current study adopted Asian-modified CKD-EPI equation instead of C-MDRD equation to evaluate GFR. C-MDRD formula had been used in previous investigations in China, which was modified on the basis of CKD population in patients with eGFR < 60 mL/min//1.73 m^2^ and in the elderly^3, 31^. But C-MDRD formula tends to underestimate normal kidney function and overrate the prevalence of CKD^35^. Asian-modified CKD-EPI equation was developed from subjects regardless of kidney diseases and it was more precise and accurate than C-MDRD equation, especially at a higher eGFR^36–39^. For example, in Romania the prevalence of CKD was 0.95% by C-MDRD equation whereas 0.64% by CKD-EPI equation in those aged between 18 and 44^3^. In this study, the ratio of eGFR below 60 mL/min//1.73 m^2^ evaluated by C-MDRD equation was twice as that by Asian-modified CKD-EPI equation. A systemic underestimation of GFR by the C-MDRD equation may account for a higher prevalence of CKD in the previous studies in China. Forth, none of the previous studies used hematuria in defining CKD according to KDIGO guideline^5, 22^. If hematuria was excluded from the definition, the prevalence of CKD markers was decreased to 1.54% in married population of reproductive age in rural China.

Hepatitis B virus (HBV) infection is highly endemic in rural China^40^. Several studies have revealed a strong association of CKD with increased morbidity and mortality of hepatitis B^18^. At present, the studies on risk factors for CKD in China seldom involve HBV infection. We found that the prevalence of CKD markers is higher in the participants with positive HBsAg than those with negative HBsAg; and importantly, positive HBsAg is an independent risk factor for CKD. The result informs that universal hepatitis B vaccination program for infants enacted in 1992 to control HBV infection might also be beneficial in controlling CKD in China.

According to the economic development level and geographical locations, Mainland China has been divided into Eastern Region, Central Region and Western Region. There have been major differences in these three regions. The Eastern Region is coastal, where local economy is developed. In the Western Region, geography is complex, natural disasters happen frequently and development lags behind. The Central Region lies inland with moderate economy development level. The Gross Domestic Product (GDP) per capita in the Eastern Region is 1.8 and 1.7 times higher than that of the Western Region and the Central Region, respectively, in 2012^41^. Different from other countries and regions where the prevalence of CKD increased with low SES and poverty^5, 8, 9, 27^, our study finds that the prevalence of CKD markers in the Eastern Region exceeds that of both the Central and the Western regions in China. Another study also found that economic development was positively associated with albuminuria in rural China^22^. There are several explanations for different prevalence of CKD markers among different geographical locations. First, rapid economic development and urbanization result in changes of life style in the Eastern rural region. The highest prevalence of hypertension and obesity stemming from modern life style may explain the highest prevalence of CKD markers in the Eastern rural region. Second, hepatitis B infection as an independent risk factor for CKD is more prevalent in the Eastern Region than that in the Central or Western regions. Third, heredity diversity may be another important factor.

Regarding gender distribution, inconsistent results have been reported. In the studies of Guangzhou, Shanghai and Mainland China, prevalence of CKD in women was all higher than that in men^22, 23, 25^. A similar female predominance had been observed in other countries^3, 8, 42^. The authors explained that the discrepancy was attributed to naturally lower eGFR in women. But in Japan, males were more susceptible to CKD due to a higher uric acid level^4^. In Taiwan, the prevalence of CKD in men was also higher than that in women^5^. In our study, the incidence of hypertension, obesity, and HBV infection, as well as the mean age of females were all lower than those of males, but the prevalence of CKD markers in females was as 2 times as that in males. Even after hematuria was excluded from the definition of CKD, the prevalence CKD markers in females remained much higher than that in males. Different congenital susceptibility to kidney damage may contribute to different prevalence of CKD markers between males and females.

This study has some limitations. First of all, according to KDIGO guideline, CKD should have indicators of kidney damage over three months. In this research, all the indicators of CKD were tested from single measurement, because it was extremely difficult to conduct repeated examination in such a large-scale investigation. Lack of confirmatory dipstick urinalysis and demonstrated chronicity of eGFR below 60 mL/min//1.73 m^2^ may lead to remarkedly overrated CKD prevalence^42^. Although some authors would believe that most of the previous studies using single measurement for serum creatinine and dipstick urinalysis might have been equally affected^43^, this limitation would result in variation in prevalence of CKD across studies. The actual prevalence of CKD in married population with fertility desire in rural China would be even lower than that in our study. Second, applying dipstick urinalysis as screening experiment might have missed diagnosis of kidney damage^20, 44^, thus underestimating the prevalence of CKD. However, in the nationwide investigation, dipstick urinalysis has been used as the most convenient, feasible and cost-effective tool to identify kidney damage, especially in rural areas. Dipstick urinalysis is sensitive enough to detect urinary protein equivalent to microalbuminuria^45^. Glomerulnephtitis remains the most common chronic kidney disease and the leading cause of end-stage renal disease in China^46^. So dipstick urinalysis, rather than urinary albumin to creatinine ratio, was selected to diagnose hematuria and proteinuria^47^. The higher prevalence of hematuria among young women might be from renal or urological origin, but not necessarily caused by CKD, which requires further investigation. Third, there was possible potential selection bias that would underestimate the prevalence of CKD. The population with serious CKD might not enroll in the survey because of having no fertility desire. Unmarried people in rural China were not included, which might be another major limitation in the current research. Forth, fasting blood glucose was measured only for women, which was another major limitation. The association of diabetes with CKD prevalence stratified by gender requires further investigation.

Despite the limitations, with random sampling and a 3-million sample size, this study had a high power that the power of CKD markers (by Asian modified CKD-EPI equation) and CKD markers (by Asian modified CKD-EPI equation and excluding hematuria) between males and female was 100%. For the first time, our study has reported the prevalence of CKD markers in married population with the mean age of 27 years in rural China. Our study also highlights variation in the prevalence of CKD across regions and potential factors attributed to this variation. In rural China, the prevalence of CKD markers in married population with fertility desire is low. Different from other studies in which the prevalence of CKD was higher in people with low socioeconomic status, a higher prevalence of CKD markers is observed in the developed Eastern Region. Hypertension, obesity, positive HBsAg, female, age and living area were independent risk factors for CKD. These results indicate that economic development and changing life style have affected the epidemiology of CKD. Better understanding and management of risk factors for CKD is critical to prevent this disease.
